# Solid Nanomedicines of Nifurtimox and Benznidazole for the Oral Treatment of Chagas Disease

**DOI:** 10.3390/pharmaceutics14091822

**Published:** 2022-08-29

**Authors:** Miriam Rolon, Eustine Hanna, Celeste Vega, Cathia Coronel, Maria Auxiliadora Dea-Ayuela, Dolores R. Serrano, Aikaterini Lalatsa

**Affiliations:** 1 Centro para el Desarrollo de la Investigacion Científica (CEDIC), Manduvirá 635 entre 15 de Agosto y O’Leary, Asuncion 1255, Paraguay; 2 Biomaterials, Bio-Engineering and Nanomedicines (BioN) Laboratory, Institute of Biomedical and Biomolecular Sciences, School of Pharmacy and Biomedical Sciences, University of Portsmouth, White Swan Road, Portsmouth PO1 2DT, UK; 3 Departamento de Farmacia, Facultad de Ciencias de la Salud, Universidad CEU Cardenal Herrera, Edificio Seminario s/n, Moncada, 46113 Valencia, Spain; 4 Department of Pharmaceutics and Food Technology, Instituto Universitario de Farmacia Industrial (IUFI), School of Pharmacy, University Complutense de Madrid, Plaza Ramón y Cajal s/n, 28040 Madrid, Spain; 5 School of Pharmacy and Biomedical Sciences, John Arbuthnot Building, Robertson Wing, University of Strathclyde, 161 Cathedral Street, Glasgow G4 0RE, UK

**Keywords:** chagas disease, trypanosomiasis, self-nanoemulsifying drug delivery systems (SNEDDS), nifurtimox, benznidazole, silicon dioxide (silica), solid self-nanoemulsifying drug delivery systems (solid-SNEDDS)

## Abstract

Chagas disease (CD) is a parasitic zoonosis endemic in Central and South America affecting nearly 10 million people, with 100 million people at high risk of contracting the disease. Treatment is only effective when received at the early stages of the disease and it involved two drugs (nifurtimox (NFX) and benznidazole (BNZ)). Both treatments require multiple daily administrations of high doses, suffer from variable efficacy and insufficient efficacy in chronic CD, many side effects, and a very long duration of treatment that results in poor compliance, while combined available therapies that lead to reduced duration of treatment are not available and polypharmacy reduces compliance and increases the cost further. Here we present self-nanoemulsified drug delivery systems (SNEDDS) able to produce easily scalable combined formulations of NFX and BNZ that can allow for tailoring of the dose and can be easily converted to oral solid dosage form by impregnation on mesoporous silica particles. SNEDDS demonstrated an enhanced solubilisation capacity for both drugs as demonstrated by flow-through studies and in vitro lipolysis studies. High loading of SNEDDS to Syloid 244 and 3050 silicas (2:1 *w*/*w*) allowed clinically translatable amounts of both NFX and BNZ to be loaded. Tablets prepared from NFX-BNZ combined SNEDDS loaded on Syloid 3050 silicas demonstration near complete dissolution in the flow through cell apparatus compared to NFX and BNZ commercial tablets respectively (Lampit^®^ and Rochagan^®^). NFX-BNZ-SNEDDS demonstrated nanomolar efficacy in epimastigotes and amastigotes of *T. cruzi* with acceptable selectivity indexes and demonstrated enhanced survival and reduced parasitaemia in acute murine experimental models of CD. Thus, the results presented here illustrate the ability for an easily scalable and personalised combination oral therapy prepared from GRAS excipients, enabling treatment access worldwide for the treatment of CD.

## 1. Introduction

Many infectious and parasitic diseases have earned the label of “neglected” because healthcare markets in the afflicted countries are insufficient to attract pharmaceutical industry funding in research and development. These involve African Trypanosomiasis, Chagas disease (CD), dengue, leishmaniasis, leprosy, lymphatic filariasis, malaria, onchocerciasis, schistosomiasis, and tuberculosis. CD is a parasitic zoonosis endemic in most mainland countries of Central and South America affecting nearly 10 million people, while more than 100 million people are at high risk [1,2]. CD is caused by infection with the protozoa *Trypanosoma cruzi* (*T. cruzi*) transmitted via the triatomine bite wound or mucous membranes [3]. CD has been estimated to have a mortality rate that can vary between 1.5–51% and a recent meta-analysis indicated that the relative risk of mortality is 1.74 (95% CI 1.49–2.03) and the attributable risk percent was 42.5% regardless of their clinical presentation [4]. Infection is also possible through food, blood or organ transplants as well as vertical transmission from an infected mother during birth [2,3].

CD presents as an acute and chronic disease. The acute phase lasts weeks to months and if patients are effectively treated with recommended protocols involving nifurtimox (NFX) and benznidazole (BNZ) for 90 days, the disease will not progress to the chronic phase [2]. Combined therapies have been shown to reduce the duration of treatment required [5] but are not available and polypharmacy only reduces compliance further while increasing the cost of therapy. Experimental and clinical studies indicate that benznidazole exposure could be reduced without loss of efficacy and combination therapy with compounds targeting different pathways has been suggested to improve efficacy [5]. Although acute phase symptoms will eventually subside even if not treated or managed, the patient will enter a chronic stage, which cannot be cured but only be managed with chemotherapy to slow down the progression of the disease. The chronic stage can take months to years to occur and 20–40% will develop life-threatening heart (congestive heart failure, sudden cardiac arrest) and/or digestive disorders (dysphagia due to enlarged oesophagus and abdominal pain and constipation due to enlarged colon).

No vaccine is currently available, although parasite DNA vaccines [6] are under development, and no drug provides satisfactory treatment for CD. Nifurtimox (NFX, 8–10 mg/kg daily over 90–120 days) and benznidazole (BNZ, 5–7 mg/kg orally in two divided doses over 30–60 days) are nitroheterocyclic compounds and the only licensed treatments for CD and they are frequently used in [7].

Nifurtimox is available commercially as 120 mg tablets (Lampit©, Bayer HealthCare) and recent Phase I studies have shown that newer 30 mg tablets of NFX are bioequivalent in infected chronic patients to 120 mg tablets suggesting the efficacy of NFX at low doses [2]. NFX is intracellularly reduced to unstable nitro-anions of NFX followed by redox cycling, yielding toxic superoxide anions and hydrogen peroxide which react with the nucleic acids of the parasite causing significant DNA damage. Prolonged action of these metabolites is maintained as *T. cruzi* lacks detoxification mechanisms for oxygen metabolites and thus is more sensitive to oxidative stress. NFX can completely inhibit parasite growth in the nanomolar range (1–10 µg mL^−1^) over 3 days [8] and is active against intracellular amastigotes. Entrapping NFX in nanoparticles has resulted in lower IC_50_ than the free drug, increased trypanocidal activity on intracellular amastigotes and reduction of toxicity that is observed at concentrations above 20 µg mL^−1^ [9]. NFX is poorly soluble and poorly absorbed (has a logP of 0.77 [1]), requires three times a day administration, possesses a short half-life and has a high volume of distribution allowing it to distribute in cardiac, skeletal and smooth muscles as well as glial cells considering the disseminated nature of the disease. NFX is stable under tropical conditions and when protected from light for up to 30 days at 50 °C and relative humidity (RH) of 100% [10].

Benznidazole (BNZ) is a triazole compound that elicits covalent modification of macromolecules by nitro-reduction intermediates while it can also inhibit protein and RNA synthesis in trypanosome parasites [11]. BNZ is active in the nanomolar range (3–6 µg mL^−1^) with toxicity occurring above a concentration of 20 µg mL^−1^ [12]. BNZ is less potent than NFX both in epimastigotes and intracellular parasites but possesses good oral bioavailability (92%) and better solubility than NFX in distilled water or simulated gastric and enteric fluids (0.2–0.4 mg mL^−1^) [1].

The ideal drug for CD treatment according to the World Health Organisation (WHO) should have the following characteristics: parasitological cure during the acute and chronic phases, efficacy in one dose or a few doses, low cost, absence of side effects or teratogenic effects, and no induction of resistance [2]. Oral therapy for CD is beneficial as the parasite resides within the gastrointestinal tract and can allow for some degree of local treatment alleviating digestive effects. Frequency of administration and duration of treatment (>90 days) results in poor compliance and reduced efficacy of treatment in the acute phase remains a challenge in the treatment and management of CD. Adverse effects of these drugs are dose-dependent and result in treatment cessation in 12–18% of patients [13]. Current research is undertaken to develop NFX (Phase III) and BNZ paediatric formulations [14], and to reduce the frequency of administration by developing NFX sustained release formulations [15] and BNZ extended-release tablets [16,17]. Additionally, recent studies to increase their oral bioavailability have also been reported in the literature such as microcrystals [18], amorphous solid dispersions [19], cyclodextrin complexes [20] and polymersomes [21] for BNZ and nano-enabled mostly polymeric or lipidic (liposomes, solid lipid nanoparticles) strategies for NFX [1]. However, although recent studies have shown that a combination of NFX and BNZ can reduce the duration of treatments to 2 months [22,23], there are no combined oral and ideally solid dosage forms available.

Our previous recently published studies demonstrated the development of orally bioavailable amorphous amphotericin B formulations [24] for CD and buparvaquone self-nanoemulsifying drug delivery systems (SNEDDS) and solid SNEDDS for the treatment of VL with the ability to elicit bioavailable buparvaquone levels and able to reduce parasite load in the spleen and to an extent in the liver [25,26]. In this study, optimised cost-effective and readily scalable from generally regarded as safe (GRAS) material SNEDDS loaded with NFX, BNZ and both NFX and BNZ that can be prepared into solid SNEDDS and directly compressed into solid dosage forms for the treatment of CD. Prepared SNEDDS were able to enhance the solubilisation capacity of NFX and BNZ and provide an easy method to combine them into oral liquid and solid clinically translatable formulations with a predicted shelf-life of 1 and 2 years respectively under refrigeration. Combined SNEDDS with low levels of NFX and BNZ show excellent selectivity for *T. cruzi* compared to fibroblasts and show a favourable target product profile for clinical translation, while they were able to statistically increase survival and reduce parasitaemia in a murine model of acute infection.

## 2. Materials and Methods

### 2.1. Materials

Nifurtimox (>98%, HPLC, 043M4717V) and BNZ (>97%, HPLC, MKBV6765V) and pancreatin (porcine, X4, USP, Batch: 1001987024) and bile extracts (porcine, Batch: MKBQ8333V) were obtained from Sigma Aldrich (Gillingham, UK). Labrasol (Caprylocaproyl macrogol-8 glycerides), Labrafil M1944 CS (oleyl macrogol-6 glycerides) and Capryol 90 (Propylene glycol monocaprylate, type II NF) were donated from Gatefosse (Alpha Chemicals, Berkshire, UK). Mesoporous silicas, Syloid XDP 3050 (Batch: 1000287532) and Syloid 244 (Batch: 1000292369), were donated from Grace (Grace GmbH & CO. KG (Worms, Germany). Eudragit FS 30D (30% *w*/*w* dispersion) and Eudragit NM 30D (30% *w*/*w* dispersion) were donated from Evonik Industries GmBH & Co (Frankfurt, Germany), while Eudragit RS PO and Eudragit RL PO were obtained from Degussa GmbH (Madrid, Spain). Microcrystalline cellulose (Avicel PH200), Povidone K 90 (Kollidon 90F, polyvinylpyrrolidone) and croscarmellose sodium (Primellose) were pharmaceutical grade and obtained from FMC corporation (Madrid, Spain), BASF plc (Cheshire, UK) and DFE Pharma GmbH & Co. KG (Gock, Germany) respectively. All other chemicals employed in the preparation of the buffers were of ACS reagent grade and were used as supplied from Panreac (Madrid, Spain). Solvents were of HPLC grade and were purchased from Scharlab (Madrid, Spain). All chemicals were used as received. Cell culture media were purchased from Sigma-Aldrich (Madrid, Spain).

### 2.2. Quality by Design (QbD) Studies for Optimising NFX and BNZ SNEDDS

We have previously optimised orally bioavailable SNEDDS for poorly soluble antiparasitic drugs (e.g., buparvaquone) using ternary diagrams [25] comprising of Labrasol:Labrafil 1944CS:Capryol 90 (6:3:1 *w*/*w*). In this QbD study, we wanted to limit the amount of Labrasol included and the following ranges were utilised to guide the QbD process to ideally yield a micro-emulsion Type III according to the lipid-based formulation classification system [27]. Thus, a range of Labrasol between 30–50% *w*/*w*, Labrafil 1944CS between 10–30% *w*/*w* and Capryol 90 between 20–40% *w*/*w* was screened. The globule size after dilution (1 in 400 *w*/*w*) was used as the only critical quality attribute (CQA) to optimise the SNEDDS. Drug loading was not undertaken due to the cost of the APIs and as optimal formulations were able to solubilise more than 20 mg g^−1^ of BNZ and 5 mg g^−1^ of NFX thus being able to elicit formulations that can be used both preclinically and clinically. Similarly, SNEDDS were optimised only for NFX as this is the API that is harder to solubilise in aqueous media.

A D-optimal mixture design was employed for systematic optimization using Design Expert^®^ software version 10.0.1 (M/s Stat-Ease, Minneapolis, MN, USA), selecting the Critical Material Attributes (CMAs) in order that the total amounts of Labrasol (X1), Labrafil M1994 (X2) and Capryol 90 (X3) was 1 g. Excipient ratios were selected as stated above. A total of sixteen formulations were prepared considering the points on vertices, edges and the interior surface of the selected mixture design with five replicates.

Table 1 summarises the design matrix along with levels of CMAs selected from the pseudoternary phase diagrams with maximal area for the nanoemulsion region. The three excipients were mixed and placed in a bath sonicator (150 watts, Ultrasons-H, J. P. Selecta, Barcelona, Spain) at 37 °C for 20 min to create a homogenous mixture. Each system was diluted 1 in 400 *w*/*w* with deionised water (pH 6.5 ± 0.1) and the particle size as the CQA was measured using a Zetatrac (Microtrac, MTB Espana, Tres Cantos, Spain) equipment in duplicate [28,29,30].

Mathematical modelling of the obtained experimental data fitted with a quadratic model was carried out by multiple linear regression analysis (MLRA). Only the statistically significant coefficients (*p* < 0.05) were considered in framing the polynomial equations, and the model was evaluated by analysing the *p*-values, coefficient of correlation (R^2^) and predicted residual sum of squares (PRESS). Response surface analysis was carried out employing 2D and 3D plots for understanding the relationship among the CMAs on the formulation of CQAs. Formulation optimisation was conducted by numerical optimization and considering the desirability function followed by the demarcation of the optimized formulation in the overlay plot design space. Validation of the QbD methodology was conducted by comparing the predicted responses with the observed ones with the help of linear correlation plots and residual plots.

The optimised formulation was determined to be Labrasol: Labrafil 1944CS: Capryol 90 50.00: 10.12: 39.88 (*w*/*w*) predicted to yield a droplet size of 0.51 µm (good desirability 0.828). The experimental particle size was 0.623 ± 0.102 µm, which is within the predicted range.

### 2.3. Preparation of Drug Loaded SNEDDS (NFX-SNEDDS, BNZ-SNEDDS) and Drug Loaded SNEDDS Capsules

NFX (2 mg) and BNZ (10 mg) were dissolved in Labrasol (0.5 g) and bath sonicated at 37 °C for 25 min (150 watts, Ultrasons-H, J. P. Selecta, Barcelona, Spain) to ensure complete dispersion. Labrafil 1944CS (0.1 g) and Capryol 90 (0.4 g) were then added and SNEDDS were left in a waterbath at 37 °C under shaking for 24 h (Memmert waterbath WNB 45, Schwabach, Germany). Blank SNEDDS were produced using this method without adding drugs. Drug loading was quantified using the isocratic HPLC as below after adequate dilution with mobile phase. Hydroxypropyl-methylcellulose capsules (Size 1, 22 mm, Capsugel^®^, Lonza, UK) were loaded with SNEDDS (0.25 g) using an analytical balance (Mettler Toledo AG104 microscale, Barcelona, Spain) [25].

### 2.4. Drug Loading and Drug Quantification Using High-Performance Liquid Chromatography (HPLC)

NFX and BNZ were analysed using an isocratic and gradient HPLC method. The isocratic method was modified from a previously published method [31] and the mobile phase consisted of methanol: sodium acetate (10 mM, pH 3) (42:58 *v*/*v*) mixtures filtered via 0.45 µm filters (Supor© 450, 47 mm, Pall Life Sciences, Madrid, Spain) under vacuum. Drug stocks (1 mg mL^−1^ in DMSO) were used to prepare diluted stocks between 0.03–100 µg mL^−1^, using the mobile phase. The HPLC comprised of a Jasco DG-2080-53 Degasser attached to a PU-1580 Pump, an AS-2050 autosampler and a UV1575 UV/Vis detector set at 395 nm for NFX and 313 nm for BNZ. Standards and samples were eluted at 1 mL min^−1^ using a Phenomenex Hypersil BDS C18 column (250 × 4.6 mm, 5 μm) and the injection volume was set at 20 μL. A linear calibration curve between 0.03 to 100 μg mL^−1^ was produced for NFX with a retention time of 4.24 min and between 0.03 to 50 μg mL^−1^ was produced for BNZ with a retention time of 3.95 min.

For the gradient method, a Phenomenex Onyx Monolithic C18 column (10 + 100 × 4.6 mm, 5 μm) was eluted at 1 mL min^−1^ at 25 °C attached to an Agilent HPLC system (Agilent Technologies, Cheadle, UK) equipped with an 1100 series degasser, quaternary pump, autosampler, and column heater, and attached to an Agilent 1100 series PDA detector. The ratio between mobile phase A (sodium acetate, 10 mM, pH 3 ± 0.1) and B (methanol) in terms of % of mobile phase B is: 0–5 min 30% B, 5–15 min 70% and 15–16 min 30%. The injection volume was set at 40 µL and detection was performed at 395 and 313 nm for NFX and BNZ respectively. A linear calibration curve was obtained between 0.1–100 μg mL^−1^ was produced for BNZ with a retention time of 4.27 min and between 0.1 to 100 μg mL^−1^ was produced for NFX with a retention time of 8.97 min.

### 2.5. Measurement of Particle Size and Zeta Potential of Prepared SNEDDS

Particle size for QbD studies was analysed using a Zetatrac laser diffraction particle analyser at 25 °C at a fixed 90° angle controlled using the Microtrac Flex version 10.5.0 software (U2493ZS, Microtrac, MTB Espana, Tres Cantos, Spain) after diluting SNEDDS 1 in 400 *w*/*w* with de-ionised water (pH 6.5 ± 0.1) [30]. The standard operating procedure (SOP) used for sample analysis involved that prior to every sample a set zero was run, analysis time was set to 60 s and 5 measurements were performed per run, while all measurements were run in triplicate and presented as mean ± SD. Particle size and zeta potential were also measured as previously described using a Nano-ZS Zetasizer (Malvern Instruments, Worcestershire, UK) [32,33,34,35].

### 2.6. Morphology of NFX-SNEDDS and BNZ-SNEDDS (TEM)

A drop of the aqueous diluted SNEDDS (1 in 400 *w*/*w*) was placed on a Formvar/Carbon coated grid (F196/100 3.05 mm, mesh 300, TAAB Labs Ltd., Berks, UK) prior to staining with 1% uranyl acetate and transmission electron microscopy (TEM) imaging using a Jeol JEM 1400 TEM microscope (Welwyn Garden City, UK) was used to image the particles as previously described [32,34,36]. Digital images were processed using an AMT (digital) camera.

### 2.7. Preparation of Drug Loaded Solid SNEDDS

To prepare solid SNEDDS, drug-loaded SNEDDS were adsorbed on mesoporous silica (Syloid 244 FP and Syloid XDP 3050) using a mortar and pestle and gentle mixing. Blank SNEDDS were loaded initially with different weight ratios on both silicas to produce SNEDDS: silica 1:1, 1:2, 1:3, 2:1 and 3:1 *w*/*w* ratios and the angle of repose, Carr’s index and Hausner ratio was measured (British Pharmacopoeia BP 2020, Volume V, Appendix XVII N) (please see Supplementary Materials SI-1.0 for method) [25,37]. The *w*/*w* ratio of SNEDDS to mesoporous silica that would provide the highest loading with better flow properties would be selected and was shown to be 2:1. Based on this optimum ratio, NFX-SNEDDS and BNZ-SNEDDS were loaded on both types of mesoporous silica to results in solid SNEDDS as per Table 2. Hydroxypropyl-methylcellulose capsules (Size 1, 22 mm) were loaded with SNEDDS (0.1 g) using an analytical balance (Mettler Toledo AG104 microscale, Barcelona, Spain) [25].

### 2.8. Manufacture of Tablets by Wet Granulation of Drug Loaded Solid SNEDDS

SNEDDS (blank or drug loaded, 5 g) were mixed in a mortar and pestle with microcrystalline cellulose (MCC, 4 g) and croscarmellose sodium (CCS, 1 g) and 5 mL of 1% polyvinylpyrrolidone in ethanol (PVP, Kollidon 90F) and mixed till a homogenous paste was produced. Mixtures were sieved (1.19 mm sieve, Labmas, Filtra Vabration, Madrid, Spain) to produce granules and when Syloid 244 silica were used they were also sieved to remove fines (0.5 mm sieve) and then were dried at 40 °C (Memmert, Schwabach, Germany) over 30 min. Granules moisture was measured using an infrared moisture balance (LP16, PM100, Mettler Toledo, Barcelona, Spain) by placing 0.5 g of granules in an aluminium sample pan after heating to 100 °C over 2 min. Granules (0.4 g) were compressed using a 12 mm punch and a tablet press (model B/30, 22o, J. Bonals, Barcelona, Spain) using 10 Newton of force. Press and die were brushed with magnesium stearate to prevent the table from sticking to the press.

Tablets if needed were manually coated by dipping in Eudragit RSPO and Eudragit RLPO ethanolic solutions (2 g in 10 mL) and then left to dry on non-stick paper vertically at 40 °C overnight and then dipped again and left to dry at 40 °C over 6 h horizontally and then dipped again and left to dry at 40 °C over 6 h horizontally on the opposite side. Tablets were then sealed in plastic sleeves and stored in a silica desiccator at least overnight. These coatings are designed to provide time-dependent pH independent sustained release.

### 2.9. Morphology of Solid SNEDDS Using Scanning Electron Microscopy (SEM)

Solid SNEDDS were exposed in simulated gastric fluid with no pepsin (0.1 M HCL, pH 1.2) for 90 min and then dried at 40 °C for 24 h prior to being mounted on a glass slide that was mounted on a standard SEM sample holder and fixed on a brass/aluminium stub using double-sided carbon impregnated adhesive discs. The sample was then sputtered coated with a conducting gold-palladium (10 nm, 60% gold–palladium) coating using an SEM coating system for 2 min at 30 mA (Quorum Q 15ORES, Quorum Technologies Ltd., Lewes, U.K., deposition range: 0–80 mA, deposition rate: 0–25 nm min^−1^, sputter timer: 0–60 min, vacuum pump: 50 L min^−1^, room temperature) before being viewed and photographed under a range of magnifications under high vacuum using a JEOL JSM-6060LV (Jeol, Welwyn Garden City, UK) scanning electron microscope [38].

### 2.10. In Vitro Lipolysis of SNEDDS

In vitro, lipolysis experiments were performed as previously described [25]. The lipolysis medium contained bile salts (5 mM) and phosphatidylcholine (1 mM), which simulated fasting conditions in the gastrointestinal tract (GIT), while the digestion buffer (200 mL) was composed of sodium chloride (150 mM) and Trizma maleate (2 mM) at pH 6.5 ± 0.05 [25]. A low buffer concentration was chosen to ensure that ionized fatty acids are able to change pH, while a buffer with low capacity was selected to allow the released fatty acids (FA) to reduce the pH and thus trigger the addition of sodium hydroxide [25] The lipase suspension was prepared by suspending pancreatin (16.6 g) in 110 mL of Millipore water at 37 °C and thoroughly mixing prior centrifugation (4000 rpm, 7 min at 37 °C). The pH of the supernatant was adjusted to 6.5 using 1 M sodium hydroxide, and 100 mL of the supernatant was used to initiate the lipolysis. To avoid denaturation, the preparation of the pancreatin suspension never exceeded 15 min. For the in vitro digestion study, the lipolysis medium (300 mL) was pre-warmed at 37 °C, prior to the addition of NFX and BNZ loaded SNEDDS (0.3 g) and adjusting the pH to 6.5 ± 0.01 with 1 M sodium hydroxide. Lipolysis was initiated by adding 100 mL of the freshly prepared lipase suspension. Ca^2+^ solution (0.5 M) was added at a dispensing rate of 0.045 mM (90 μL) per min (NE-1000 programmable single-syringe pumps, World Precision Instruments Ltd., Hertfordshire, UK). Throughout the study, the pH was kept constant at 6.5 ± 0.01 (Accumet AB200 equipped with an accuTupH rugged bulb, Fisher Scientific, Loughborough, UK) by the addition of 1 M sodium hydroxide. In this respect, the number of OH^−^ ions present in the volume of the titrant can be equated with the FA liberation caused by lipolysis. Samples (30 mL) were withdrawn at times of 0, 5, 15, 30, 60 after the addition of the lipase suspension. Samples were frozen using dry ice prior to ultracentrifugation at 40,000 rpm at 4 °C (CP100NX, Hitachi Koki Co. Ltd., Milton Keynes, UK). The amount of NFX and BNZ present in the supernatant and in the pellet was quantified after their appropriate dilution with methanol or isocratic HPLC mobile phase using HPLC as described above.

### 2.11. Solid SNEDDS Tablet Characterisation

Tests were undertaken only with blank solid SNEDDS tablets. Friability testing was carried out according to the British Pharmacopoeia (2021, Appendix XVII) [39] using ten SS2 tablets (Table 2) that were overcoated with Eudragit RSPO and PharmaTest Type PTF friability tester (100 rpm, Pharmatest, Hainburg, Germany) that were weighted after dusting at onset and at the end of the experiment. Tablets without coating did not pass the friability test. Tablet disintegration was undertaken for uncoated SS1 and SS2 tablets as well as Eudragit RSPO and RLPO SS1 and SS2 tablets using PharmaTest Type PTZ1 disintegration tester (Pharmatest, Hainburg, Germany) using simulated gastric fluid without pepsin (pH 1.2 ± 0.05, 37 °C). Tablet hardness for uncoated SS1 and SS2 tablets as well as Eudragit RSPO and RLPO SS1 and SS2 tablets was tested using a PharmaTest PTB311 tester (Pharmatest, Hainburg, Germany).

### 2.12. Dissolution Studies for Filled Capsules and Tablets

Dissolution media were prepared as described in the British Pharmacopoeia (2021, Volume V, Appendix XIIB) [40]. The dissolution of NFZ-SNEDDS, BNZ-SNEDDS and NFZ-BNZ SNEDDS (2, 10 and 2 and 10 mg g^−1^ respectively, 0.25 g) or solid SNEDDS (0.1 g) loaded in capsules (Hydroxypropyl-methylcellulose capsules (Size 1, 22 mm, Capsugel^®^, Lonza, UK) was studied in the flow-through cell dissolution apparatus in an open-loop configuration with a cell of an internal diameter of 22.6 mm (USP, Apparatus IV) at various pH levels [simulated gastric fluid (SGF) without enzyme (pH1.2, 1 h) and phosphate buffer (pH6.8, for remaining time), 37 ± 5 °C, flow rate = 10 mL min^−1^] as previously described [25,28]. The quantity of drug solubilised at any time point was analysed using the isocratic HPLC method and expressed as a % of total amount of drug in the tested formulations. The powder cake remaining in the flow through cell at the end of the dissolution study for the commercial 120 mg NFX tablets (Lampit^©^) and 100 mg BNZ tablets (Rochagan^®^) were bath sonicated with 85 mL of methanol at 37 °C for 1 h prior 1 in 100 *v*/*v* dilution with methanol and HPLC analysis. All experiments were run in triplicates.

### 2.13. Accelerated Stability Studies of Liquid and Solid SNEDDS

Stability studies of liquid and solid SNEDDS (NFX SNEDDS, NSS1, NSS2, NFX and BNZ SNEDDS, NBSS1, NBSS2) were undertaken over five temperatures (25, 40, 60, 70, 80 °C) and analysed for drug content at various time points [41]. Uncovered HPLC vials were placed in test stability Amebis^®^ chambers (Amebis Ltd., Dunshaughlin, Co., Meath, Ireland) filled with Silica Gel 3–6 mm with an indicator sealed with a sensor cap. Four vials of each formulation for every temperature and time point were prepared (3 to be tested and a spare). Samples were removed from ovens on appropriate days and drug content was quantified using HPLC.

Stability modelling was performed using ASAP*prime*^©^ software version 5 (FreeThink Technologies, Inc., Branford, Connecticut, CT, USA). The method used was “potency without relative humidity (RH)”, which focuses on the remaining amount of NFX and BNZ with variation in assessed temperatures. The degradation rate of NFX and BNZ was calculated by fitting the percentage of drug degraded at different time points to several degradation kinetic equations (zero order, first order, second order, Avrami and diffusion). Using the degradation rate obtained for different temperatures, the Arrhenius equation was used to calculate the activation energy and estimate the effect of temperature on the degradation rates of NFX and BNZ based on Equation (1)

(1) $$K=A\;e^{\frac{-Ea}{RT}}$$

where *K* is the degradation rate (% drug degraded per day), *A* is the collision factor, *T* is the absolute temperature in Kelvin, *R* is the gas constant (1.985 cal mol^−1^ K^−1^), and *Ea* is the activation energy in cal mol^−1^ [41,42]. The best fitted degradation kinetic model was selected based on the highest R^2^ value for shelf-life prediction at a mean kinetic temperature of 4 °C (refrigerated conditions) and 25 °C considering a specification limit of 90% drug content.

### 2.14. In Vitro Trypanocidal Assay

To test the in vitro efficacy of novel formulations, a standardized protocol for screening potential drugs for the treatment of Chagas disease was followed using epimastigotes and amastigotes because trypomastigotes are unable to replicate [43]. Screening using epimastigotes enables testing directly the efficacy of drugs dissolved in dimethylsulfoxide or formulations against the parasite and amastigotes (intracellular forms) assess the ability of the drug to permeate cellular membranes and remain effective against the amastigotes form of the parasite.

#### 2.14.1. Parasites

The *T. cruzi* clone CL-B5 were kindly provided by Dr F Buckner through Instituto Conmemorativo Gorgas (Panama) and were stably transfected with the *Escherichia coli* β-galactosidase gene (lacZ) [24]. The epimastigotes were grown at 28 °C in liver infusion tryptose broth (complemented with 10% fetal bovine serum, FBS (Internegocios, Argentina), penicillin and streptomycin) and afterwards, were harvested during the exponential growth phase.

#### 2.14.2. In Vitro Epimastigotes Susceptibility Assay

The assay was performed in 96-well microplates (Cellstar, EU) with cultures that have not reached the stationary phase, as was previously described [24]. Briefly, epimastigotes were seeded at a concentration of 2.5 × 10^5^ per mL in a total volume of 200 μL. Plates were incubated at 28 °C for 72 h with NFX or BNZ (50 µg mL^−1^ in dimethylsulfoxide (DMSO); DMSO concentration in media never exceeded 0.2%) as reference drugs or the formulations (after 1 in 50 *w*/*w* dilutions in media) and lower concentrations were obtained by serial dilution. Then, chlorophenol red−β-D-galactopyranoside solution (50 μL, CPRG Roche, Indianapolis, IN, USA) was added to obtain a final concentration of 200 μM. Plates were incubated for another 4 h at 37 °C prior to being read at 595 nm. Each concentration was tested in triplicate and each experiment was performed twice separately. The efficacy of each compound was estimated by calculating the IC_50_ (drug concentration that produces a 50% reduction in parasites) and was determined by sigmoidal regression analysis (% of viable cells vs. the logarithm of the compound concentration).

#### 2.14.3. Amastigote Susceptibility Assay

The assay was performed by a colourimetric method using chlorophenol red−β-D-232 galactopyranoside (CPRG) [24]. Briefly, NCTC-929 fibroblasts (a gift from Dr Gomez- Barrio (Universidad Complutense de Madrid, Spain)) were cultured in 24-well tissue culture plates at a concentration of 2.5 × 10^3^ cells/well which was previously optimised.

NCTC-929-derived trypomastigotes were added to the monolayers at a parasite: cell ratio of 5: 1 and were incubated for 24 h at 33 °C with 5% CO_2_. To remove the extracellular trypomastigotes, the infected cells were then washed twice with PBS. The formulations were added in triplicate resulting in a final volume of 900 μL/well. Plates were incubated for 7 days at 33 °C. CPRG solution (100 μL) in 0.3% Triton X-100 was then added to obtain a final concentration of 400 μM. The colourimetric reaction was quantified by measuring optical density (OD) at 595 nm wavelength after 4 h of incubation at 37 °C. The percentage of anti-amastigote activity (*AA%*) was expressed as indicated in Equation (2):

(2) $$AA\%=\left(100-\frac{OD\;experimental\;wells}{OD\;control\;wells\;}\;\right)\times 100\%$$

Background controls (only NCTC- 929 cells) were subtracted from all values.

### 2.15. In Vivo Trypanocidal Studies (Acute Phase)

All experiments were approved and performed in accordance with the local ethical committee of the Fundacion Moisés Bertoni (PROCIENCIA-14-INV-022, CONACYT-Paraguay). Bloodstream trypomastigotes of the Y strain (ATCC 50832) were used which were harvested from *T. cruzi* infected BALB/c mice on the day of peak parasitaemia as previously described [24]. Female 4–6 week old BALB/c mice (18−20 g) were obtained from the Animal Facility of the Instituto de Investigaciones en Ciencias de la Salud, Universidad Nacional de Asuncion (UNA, Paraguay). Mice were housed according to the standards of the Committee of Animal Welfare and were kept in a room at 20–24 °C under a 12/12 h light/dark cycle and provided with sterilized water and food ad libitum. The animals were allowed to acclimatise for 7 days before the onset of the experiments. Animals were infected by intraperitoneal injection of Y strain trypomastigotes of *T. cruzi*.

The experimental protocol performed allows the analysis of the effect of the formulations on the parasite load [24]. Mice were randomly allocated into groups of six to ensure that a 50% difference in parasitic load can be detected with 80% confidence. Mice were inoculated intraperitoneally with 10^4^ trypomastigotes from the Y strain. On day 5 post-infection, parasitaemia (number of trypomastigotes mL^−1^ of blood) was quantified microscopically using the Pizzi−Brener method [44]. Only animals that demonstrated homogeneous parasitaemia were used and treated orally starting 5 days post-infection (dpi). NFX and BNZ were administered as 5% *w*/*w* suspensions in a carboxymethylcellulose (0.5% *w*/*v*) in each animal by gavage daily over 5 consecutive days. Administration volume by oral gavage for formulations never exceeded 0.2 mL. Two studies were undertaken. In the first one, we compared the negative control to mice treated with NFX (50 mg/kg/day) or BNZ (50 mg/kg/day) orally daily over 5 days. In the second one, the negative control was compared to blank SNEDDS, NFX-BENZ-SNEDDS (2.5 and 5 mg g^−1^ of formulation to achieve a dose of 25 mg/kg/day and 50 mg/kg/day respectively), BENZ-SNEDDS (5 mg g^−1^ of formulation to achieve a dose of 50 mg/kg/day) and BENZ-SNEDDS (10 mg g^−1^ of formulation to achieve a dose of 100 mg/kg/day) administered orally once daily for 5 consecutive days starting 5 days post-infection. Parasitaemia was quantified at 5, 8, 10 dpi. The percentage of parasitaemia reduction was calculated using Equation (3):

(3) $$Parasitaemia\;\%=100-\frac{PT}{PC}\times 100\%$$

where *PC* is the number of trypomastigotes per mL^−1^ of blood in the control group and *PT* is the number of trypomastigotes per mL^−1^ of blood in the treated group at the same day post-infection [24]. The mice survival rate was recorded up until the end of the acute phase (30 days post-treatment) in all the experiments.

### 2.16. Cytotoxicity Assays

NCTC clone 929 (as above) and murine J774 macrophages were used to assess the cytotoxicity of the formulations in triplicate. The NCTC clone 929 cells were grown in Minimum Essential Medium (MEM; Sigma, St. Lois, MO, USA) murine J744 macrophages in RPMI 1640 media (Sigma, St. Lois, MO, USA) supplemented with 10% heat-inactivated FBS (30 min at 56 °C) and antibiotics (100 units mL^−1^ penicillin G and 100 µg mL^−1^ streptomycin), while and cytotoxicity assays were performed as previously described [24]. Pre-confluent cells were plated in 96-microtiter plates at 3 × 10^4^ cells/well (NCTC clone 929) or 5 × 10^4^ cells/well (J774) in 100 μL of growth medium and were grown for 16 or 24 h respectively at 37 °C in a humidified 5% CO_2_ atmosphere. Afterwards, the medium was removed and the serially diluted two-fold formulations were added in 200 μL of medium for 24 h (growth controls were included) after which resazurin solution (20 μL, 2 mM for NCTC clone 929 and 1 mM for J774 cells) was added to each well. The plates were incubated for a further 3 h and the absorbance was read at 490 and 595 nm on a microplate reader (Sinergy, Biotek, VT, USA). The cytotoxicity of the formulations was measured in terms of the concentration that was able to reduce the viability of treated cells in culture by 50% compared to untreated cells in culture (CC_50_) [45].

### 2.17. Statistical Analysis

All in vitro assays consisted of triplicate cultures per each experimental condition. The mean percentage of parasites obtained in two independent experiments was used to estimate the fifty percent inhibitory concentration (IC_50_ and CC_50_). These values were calculated by the sigmoidal doses response curve adjustment using GraphPad Prism 9.0 software (GraphPad Software, San Diego, CA, USA). One-way ANOVA and a Tukey’s HSD post-hoc test and Kruskal Wallis test were used to analyse all the in vitro and in vivo test data respectively (GraphPad Prism 9.0). Statistical significance was considered at the 5% level.

## 3. Results

A quadratic fitting model was the optimal mathematical model for linking ration of GRAS oils and surfactants to the desired CQA for SNEDDS (please see Supplementary Materials Table S4). The co-efficient of the models generated for CQAs (please see Supplementary Materials Table S5) revealed medium goodness of fit of the experimental data to the selected model but able to be used to navigate the design space (R^2^: 0.74, low values of PRESS, a negative predicted R-squared which indicates that the overall mean may be a better predictor of droplet size in the current model and a ratio greater than 4 for the adequate precision indicative of signal to noise ratio).

**Figure 1:**
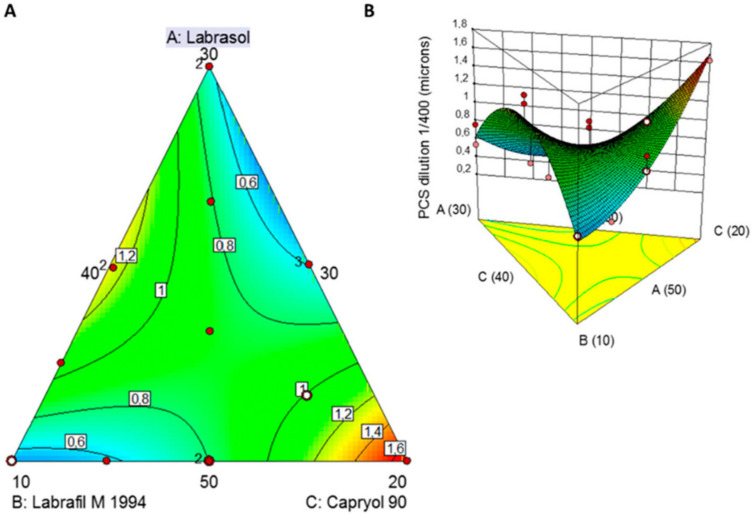
Two dimensions contour plots (**A**) and 3D response surface plot (**B**) showing the influence of Labrasol, Labrafil M 1944, Capryol 90 on the CQAs (particle size (PCS) upon 1 in 400 *w*/*w* dilution in de-ionised water (pH 6.5 ± 0.1) of SNEDDS. Red colour indicates larger particle size, while turquoise colour indicates smaller particle size).

The equation generated for the particle size in terms of actual components is shown below:

(4) $$\begin{array}{cc} Droplet\;size= & -5.50291\times 10^{-4}\times Labrasol-0.24521\times Labrafil\;M1944+0.30529\times Capryol\;90\\ & +7.02881\times 10^{-3}\times Labrasol\times Labrafil\;M1944\\ & -5.7684\times 10^{-3}\times Labrasol\times Capryol\;90\\ & -3.00141\times 10^{-3}\times Labrafil\;M1944\times Capryol\;90\; \end{array}$$

where *droplet size* is the particle size after 1 in 400 *w*/*w* dilution, *Labrasol* is the % *w*/*w* of Labrasol, *Labrafil M* 1944 is the % *w*/*w* of *Labrafil M* 1944 CS and *Capryol* 90 is the % *w*/*w* of Capryol 90 in the SNEDDS. The equation in terms of actual factors can be used to predict the response for given levels of each factor as long as levels are specified in the original units for each factor. However, this equation cannot be used to determine the relative impact of each factor because the coefficients are scaled to accommodate the units of each factor and the intercept is not at the centre of the design space. The 2D-contour plot and 3D-response surface plot (Figure 1) reveals the influence of the excipient on the particle size of the SNEDDS upon 1 in 400 *w*/*w* dilution in deionised water.

The smallest globule size was observed at low levels of Labrafil M 1994 and higher levels of Capryol 90. The globule size of all the formulations was found to be ranging between 278 nm and >1 µm. A sharp descending trend with decreased levels of Labrafil M 1994 is noted (Figure 1). The smallest globule size was particularly observed at low concentrations of lipid, high concentrations of surfactant and intermediate concentrations of cosurfactant in the formulations. This can be explained by a layer of the surfactant at the oil-water interface able to reduce the interfacial tension and stabilization of the emulsion globules. Co-surfactant (Labrafil M 1944) improves the emulsification and dispersion of the oil-surfactant particles, leading to the formation of globules in the nanometric range.

The two turquoise-coloured areas in Figure 1A indicate two optimised SNEDDS demarcated in the design space overlay plot. The first one was composed of Labrasol:Labrafil M 1944:Capryol 90 50.0:10.1:39.9 *w*/*w* which resulted in a particle size of 509 nm and the second one Labrasol:Labrafil M 1944:Capryol 90 35.4:30.0:34.6 *w*/*w* which resulted in a mean size of 471 nm.

**Figure 2:**
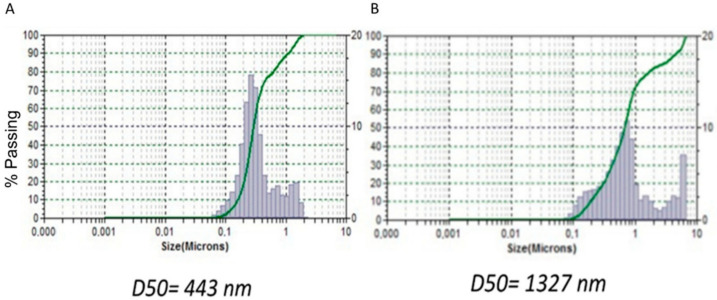
Validation of the QbD methodology for the optimised SNEDDS 1 (**A**) and 2 (**B**). *Y*-axis spans between 0–100% with labels every 10% and *X*-axis is a logarithmic scale spanning between 0–10.

Validation of the QbD methodology revealed close proximity among the predicted values of the optimised formulation 1 with experimental particle size values (Figure 2). The percent prediction error (i.e., percent bias) for the CQAs varied between −0.18% and 1.20% with overall mean ±SD of 0.509 ± 0.237. However, the experimental optimised formulation 2 revealed a much higher variability with the predicted values (1327 vs. 471 nm). For this reason, optimised formulation 1 was selected for further studies.

**Figure 3:**
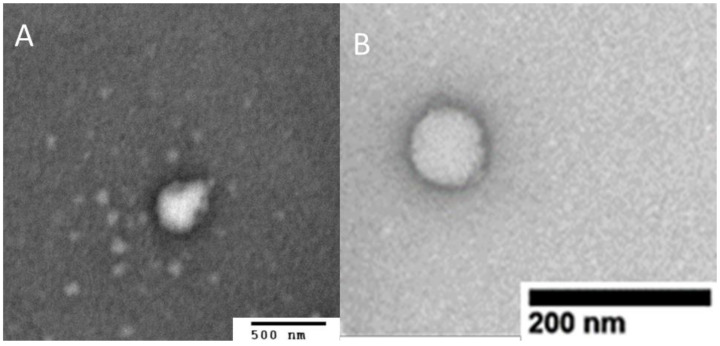
TEM images of aqueous dispersions of NFX-BNZ-SNEDDS (**A**,**B**) (1 in 400 *w*/*w*) stained with 1% uranyl acetate. Bars: 500 and 200 nm respectively.

Prepared NFX-SNEDDS, BNZ-SNEDD and NFX-BNZ SNEDDS illustrated sizes consistently below 200 nm (Table 3), good colloidal stability and spherical morphology (Figure 3). High loading of NFX (0.2% *w*/*w*, 2 mg g^−1^) and BNZ (1 and 2% *w*/*w*, 10 and 20 mg g^−1^) was possible in SNEDDS (higher than previous reports [46,47,48]) allowing them to be clinically relevant orally. Particle size of loaded formulations were significantly smaller and below 200 nm (*p* < 0.05, One-way ANOVA).

**Figure 4:**
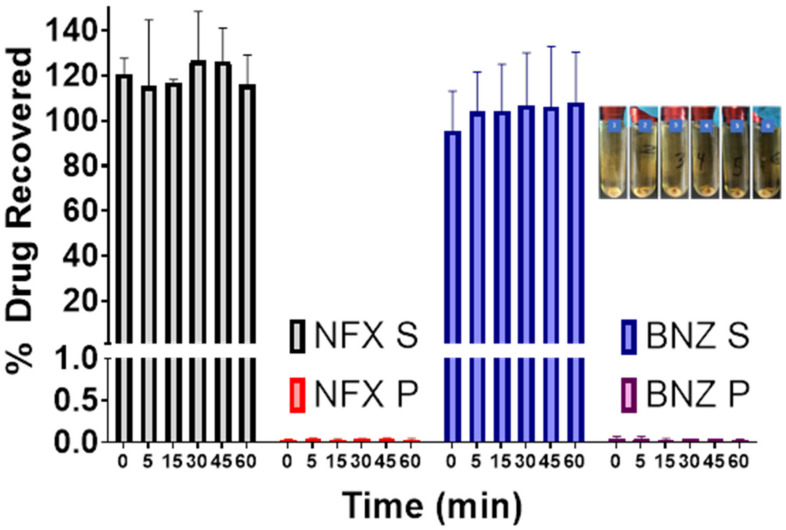
In vitro lipolysis of NFX-BNZ SNEDDS with grey and red bars illustrating the % of NFX and blue and purple bars illustrating the % of BNZ recovered in the aqueous supernatant phase (S) and pellet (P) respectively (*n* = 3). Picture shows samples after ultracentrifugation in progressive time order of 0, 5, 15, 30, 45 and 60 min.

The in vitro dynamic lipid digestion model offers information regarding drug partitioning during hydrolysis of triacylglycerides and quantifying the amount of solubilized drug in the intestinal fluids (Figure 4). Lipolysis is initiated by the addition of the lipase suspension, after which the lipolytic process is monitored as a function of time. Calcium chloride solution was added continuously to control the accumulation of fatty acids in the medium by forming insoluble calcium FA soaps, which precipitate, thus removing FA from the system and preventing accumulation [25]. Over the duration of 60 min, both NFX and BNZ remained solubilised and unprecipitated and thus available for absorption.

Both types of mesoporous silica (Syloid S244 and XDP3050) resulted in free-flowing powders with maximum SNEDDS loading when a ratio of 1:2 *w*/*w* was used respectively (please see Supplementary Materials Tables S1 and S2, and Figure S1). Higher ratios between silica and SNEDDS were not able to adsorb all the quantity of SNEDDS and were not acceptable for further manufacture of solid dosage forms (capsules or tablets). Loading of solid SNEDDS prepared is summarised in Table 4.

**Figure 5:**
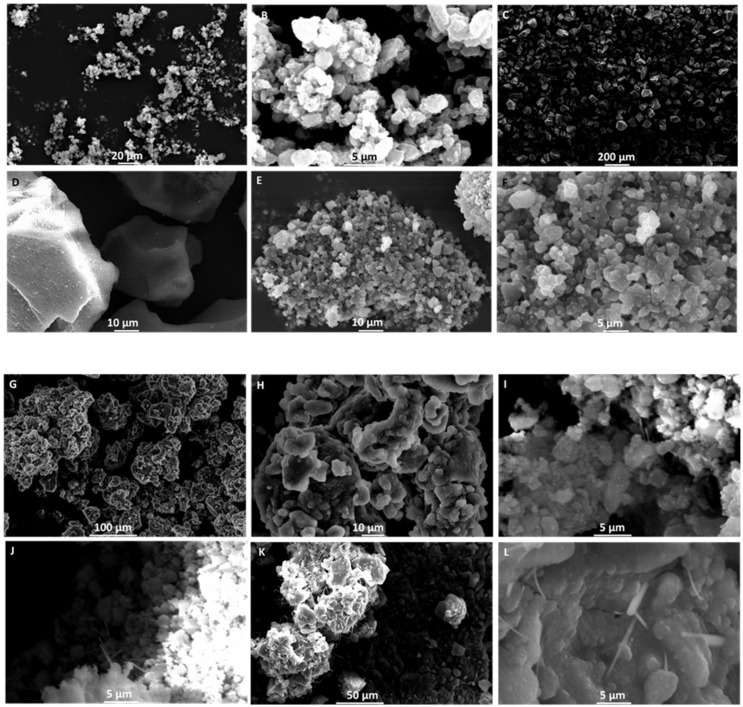
Scanning electron microscopy (SEM) images to understand the morphology of solids SNEDDS; Blank solid SNEDDS using Syloid 244 FP 2:1 *w*/*w* (SS1, (**A**,**B**), Bar: 20 and 5 µm), blank solid SNEDDS using Syloid XDP 3050 2:1 *w*/*w* (SS2, (**C**,**D**), Bar: 200 and 10 µm), NFX—BNZ SNEDDS adsorbed on Syloid 244 FP 2:1 *w*/*w* (NBSS1, (**E**,**F**), Bar: 10 and 5 µm), NFX—BNZ SNEDDS adsorbed on Syloid XDP 3050 2:1 *w*/*w* (NBSS2, (**G**,**H**), Bar: 100 and 10 µm), NBSS1 exposed in acidic media (0.1 M HCl for 90 °C) and dried ((**I**,**J**), Bar: 100 and 10 µm) and NBSS2 exposed in acidic media (0.1 M HCl for 90 °C) and dried ((**K**,**L**), Bar: 50 and 5 µm).

SEM studies indicated that SS1 (blank SNEDDS:Syloid 244 FP 2:1 *w*/*w*) powders have a smooth and slightly angular morphology with a pore size of ~3.5 µm, which match closely product specifications (free flowing particle with pores of 2.5–3.7e µm). However, the particle size distribution was wider to SS2 (blank SNEDDS:Syloid XDP 3050 2:1 *w*/*w*) particles (Figure 5A,B). SS2 particles were rough with a highly porous surface (pores of 10–25 µm), which is able to load high amounts of SNEDDS. The rough surface in combination with higher pore size explains the better flowability observed for SS2 Figure 5C,D). Both NBSS1 and NBSS2 drug-loaded mesoporous silica particles showed a reduction in pore size and NBSS2 particles were less angular than unloaded particles indicative of coating of the particle surface (Figure 5E–H). Exposing these particles to an acidic environment for 90 min (0.1 M hydrochloric acid), we observed the presence of eluted fibrils out of the long pores of the silica particles after drying and this was more abundant in the NBSS2 particles (Figure 5I–L). Based on our current experiments, it is not clear whether these produced fibres are mesoporous silica fibres prepared in static acidic media in the presence of oils and surfactants in SGF [49]. Drug release from solid SNEDDS powder formulations depends on the physical characteristics of solid adsorbents in addition to the molecular interaction between the drug, lipid/surfactant/co-surfactant mixture and the solid carrier [50,51]. The increased length of the pores may decrease access of water to entrapped drug and diminish the hydration of the formulation. Carriers with the low specific surface area may facilitate dispersion of the drug in the dissolution medium leading to faster dissolution performance, while carriers with high specific surface area (mesoporous silicas >300 m^2^/g) may not adequately disperse the drug and this may result in reduced dissolution behaviour. This has been demonstrated previously for gentamycin sulphate dispersed in Labrasol^®^ and adsorbed onto the surface of different silicate-based adsorbents [52]. The role of silanol/hydroxyl groups in the interaction with the SNEDDS can also explain the hindered release and the higher affinity for the surface of Syloid 244 can explain the release hindrance. The presence of fibrils could be a direct result of the high surface tension of the SNEDDS and the capillarity of pores within the silica. Exposing the silica particles in acidic media reduces the surface roughness and this was more visible for NBSS2 particles.

**Figure 6:**
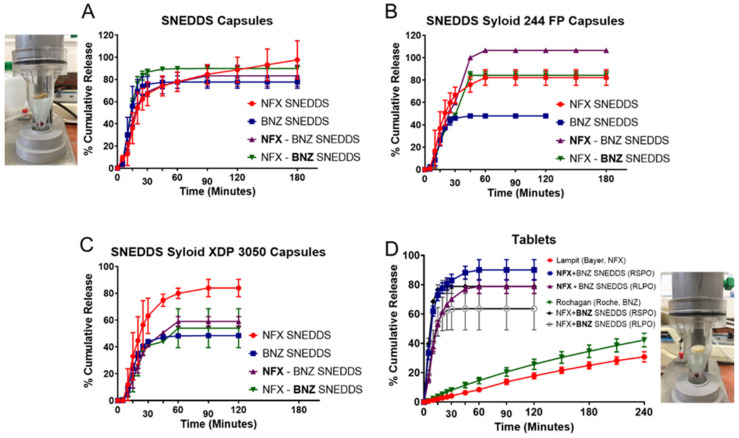
In vitro dissolution studies (Apparatus IV) of SNEDDS filled capsules (**A**), SNEDDS loaded in Syloid 244 FP (2:1 *w*/*w*) and filled in capsules (**B**), SNEDDS loaded in Syloid XDP 3050 (2:1 w.w) and filled in capsules (**C**) and tablets (**D**). Key: For combined NFX-BNZ SNEDDS (0.2% NFX and 1% BNZ combined) data, the dissolution of the drug highlighted in bold is depicted (i.e., **NFX**-BNZ SNEDDS demonstrates the release of NFX from the combination). Significant statistical comparisons; (**B**): The % cumulative release for both NFX and BNZ from both NFX-SNEDDS and BNZ-SNEDDS loaded in Syloid 244 FP is statistically significantly lower than the % cumulative release of both drugs from the NFX-BNZ-SNEDDS loaded in Syloid 244 FP (*p* < 0.05, *t*-test at all time points after 30 min), (**C**): The % cumulative release of NFX from both NFX-SNEDDS loaded in Syloid XDP 3050 is statistically significantly higher than the % cumulative release of NFX from the NFX-BNZ-SNEDDS loaded in Syloid XDP 3050 (*p* < 0.05, *t*-test at all time points after 30 min), (**D**): Commercial tablets for NFX (Lampit^®^) and BNZ (Rochagan^®^) were tested versus compressed tablets of combined NFX-BNZ SNEDDS at a 2:1 *w*/*w* loaded on Syloid XDP 3050 that were overcoated with Eudragit RS PO (RSPO) or Eudragit RL PO (RLPO). The % cumulative release for both NFX and BNZ from both types of tablets is statistically significant from % cumulative release from Lampit^®^ and Rochagan^®^ tablets (*p* < 0.05, Repeated measures ANOVA, post-hoc Tukey’s test at all time points up to 120 min).

A higher yield than 70% was obtained after wet granulation and sieving and all pre-compression powder had a moisture content below <2%. Best tablets were obtained with compression under 10 N of granules of a higher Avicel pH200 ratio to solid SNEDDS and thus a ratio of solid SNEDDS:microcrystalline cellulose (Avicel PH200): croscarmellose sodium (Primellose) of 5:4:1 *w*/*w* (please see Supplementary Materials Figure S2). Near complete release was obtained for both NFX and BNZ from liquid-filled capsules (Figure 6A), with a slower release rate observed in higher pH media (after 1 h, pH 6.8 phosphate buffer). Increasing the amount of SNEDDS loaded in Syloid silicas may decrease dissolution performance. This was evident for both NFX and BNZ, but the dissolution profile of the latter was more affected. SNEDDS loaded in mesoporous silica resulted in a slower release rate for BNZ (*p* < 0.05 at all time points after 20 min, Two-way ANOVA, Tukey’s post-hoc test, Figure 6). The nitro group of BNZ is more likely to interact with hydroxy groups of silicas. NFX were nearly completely released from both Syloid 244 FP and XDP 3050 solid SNEDDS loaded in immediate-release capsules. NFX release was hindered when formulated with BNZ using Syloid XDP 3050 silicas and this was obvious in both the filled immediate-release capsules and when granulated and compressed into tablets (*p* < 0.05, Two-way ANOVA, Tukey’s post-hoc test, Figure 6D). Commercial tablets reached less than 40% release after 4 h for both NFX and BNZ in dissolution studies using the flow-through cell (Figure 6D). Dissolution from Rochagan tablets obtained in Figure 6D remained low (14.63 ± 1.52%) after one hour in SGF, which is lower than previous reports using Apparatus I [53] and II [18]. We feel that this is attributed to delays in the disintegration of commercial tablets in Apparatus IV due to the presence of lower initial volume, compared to 900–1000 mL, and potentially a higher flow rate required than 10 mL min^−1^ than used in our experiments. Further experiments are required to understand this, but our studies remain comparable as all formulations presented were tested under the same conditions.

Only Eudragit RLPO double-coated tablets of SNEDDS loaded on Syloid XDP 3050 silica passed the friability test. The disintegration of coated and uncoated Syloid XDP3050 tablets were below 10 s, while Syloid 244 FP tablets coated, and uncoated tablets needed more than 50 s to disintegrate. All tablets prepared failed the hardness test and were too brittle. This has been previously reported and higher compression forces are used to overcome this issue (~12–20 N), which are higher than the force we have used in these experiments [54].

In vitro dissolution studies have shown that release from NFX-SNEEDS and BNZ-SNEDDS is not different from the release of each drug from combined NFX-BNZ-SNEDDS and reaches a value of 90% of cumulative release within 30 min. This indicates that both NFX and BNZ can remain solubilised in simulated gastrointestinal media. Using combined NFX-BNZ-SNEDDS loaded onto Syloid 244 FP, a higher % of cumulative release was observed compared for both drugs compared to the release of each drug from NFX- SNEDDS or BNZ-SNEDDS loaded on to Syloid 244 FP at the same ratio (2:1 *w*/*w*). This can be explained by stronger interactions of drug impregnated SNEDDS to Syloid 244 FP when each drug-loaded SNEDD is loaded individually that are abolished when drugs are present together potentially due to an intramolecular interaction (hydrogen bond) between BNZ and NFX allowing for higher affinity for the surface. However, the opposite was observed for NFZ-BNZ-SNEDDS loaded on Syloid XDP 3050, where release from combined NFX-BNZ-SNEDDS is hindered indicating that other interactions (van der Walls) are also important. BNZ release from BNZ loaded SNEDDS on both Syloid particles is hindered compared to BNZ loaded SNEDDS alone due to hydrogen bonding with silanol/hydroxyl group and this is more significant for BNZ compared to NFX. Release from compressed tablets was significantly higher than that achieved from commercial tablets of both drugs under our testing conditions (Figure 6D). High ratio of SNEDDS impregnated to silica particles (2:1 *w*/*w*) explains the immediate release profile observed. Overcoating the tablets was unsuccessful as an immediate release profile was observed and there were no statistical differences between the release profile of each drug and the type of coating.

**Figure 7:**
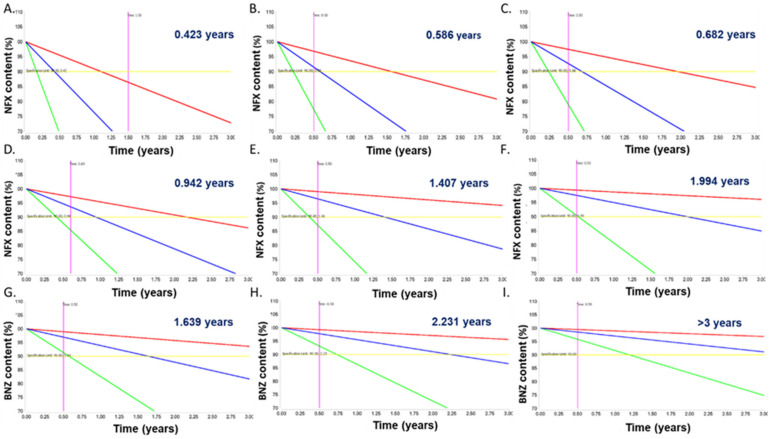
Shelf-life prediction in years of formulations under refrigerated conditions. NFX-SNEDDS (**A**), NFX-SNEDDS impregnated on Syloid 244 FP (2:1 *w*/*w*) (**B**), NFX-SNEDDS impregnated on Syloid 3050 XDP (2:1 *w*/*w*) (**C**), **NFX**-BNZ SNEDDS (**D**), **NFX**-BNZ-SNEDDS impregnated on Syloid 244 FP (2:1 *w*/*w*) (**E**), **NFX**-BNZ-SNEDDS impregnated on Syloid 3050 XDP (2:1 *w*/*w*) (**F**), NFX-**BNZ**-SNEDDS (**G**), NFX-**BNZ**-SNEDDS impregnated on Syloid 244 FP (2:1 *w*/*w*) (**H**), NFX-**BNZ**-SNEDDS impregnated on Syloid 3050 XDP (2:1 *w*/*w*) (**I**). Key: Drug highlighted in bold indicates the drug of the combined NFX-BNZ SNEDDS that the stability data are shown. Yellow line indicates the specification limit (90%). For all graphs, *Y*-axis spans between 70–110% with labels every 5% and *X*-axis spans between 0–3 years with labels every 0.25 years.

NFX and BNZ followed a first order degradation kinetics in both liquid and solid SNEDDS formulations (R^2^ > 0.9). The activation energy ranged from 18–27 Kcal mol^−1^ depending on the formulation being greater for solid SNEDDS (Table 5). The stability of NFX in the liquid SNEDDS was poor, exhibiting a shelf-life of just 0.423 years under refrigerated conditions and just over 6 days (0.019 years) at 25 °C (Figure 7A). However, the chemical stability of the drug was enhanced upon transformation into a solid formulation resulting in a shelf life for the NFX-SNEDDS Syloid 244 and 3050 XDP of 0.586 and 0.682 years under refrigerated conditions respectively (Figure 7B,C). The stability of NFX was further improved when combined with BNZ both in the liquid and the solid formulations exhibiting an increase of 2–3 fold in shelf-life (Figure 7D–F) under refrigerated conditions and 4.3 fold at 25 °C. The BNZ showed a better stability profile than NFX, being stable under refrigerated conditions for over a year in liquid SNEDDS and above 2 years when transformed into a solid SNEDDS formulation (Figure 7G–I). The enhancement in chemical stability can be related to both the physicochemical interactions between NFX and BNZ and interactions with the silanol groups exposed in both mesoporous silicas. This stability profile facilitates the clinical translation of the liquid and solid NFX-BNZ formulations.

NFX-SNEDDS, BNZ-SNEEDS and NFX-SNEDDS demonstrated nanomolar efficacy in *T. cruzi* epimastigotes (Table 6 and Table 7). SNEDDS showed excellent selectivity for *T. cruzi* compared to fibroblasts and show a favourable target product profile for clinical translation [55]. Good selectivity index (CC_50_/IC_50_) was obtained for NFX-BNZ-SNEDDS against epimastigotes as well as ~95% growth inhibition of intracellular parasites.

**Figure 8:**
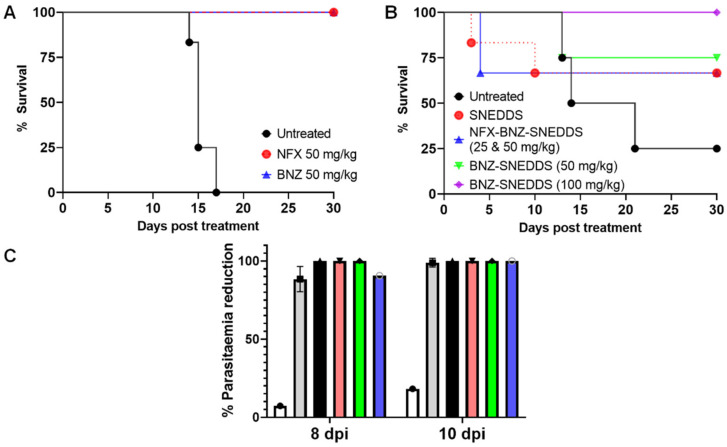
(**A**): Kaplan-Meier survival plot comparing the untreated control (black line) to oral suspensions of NFX (50 mg kg^−1^, red line) and BNZ (50 mg kg^−1^, blue line) administered orally over 5 days (*n* = 6). Survival at day 30 post-treatment is significant for both NFX and BNZ groups (*p* < 0.0001, Kruskal Wallis test, 95% level of significance). (**B**): Kaplan-Meier survival plot comparing the untreated control (black line) to orally administered blank SNEDDS (red line), NFX-BNZ-SNEDDS (25 and 50 mg kg^−1^ respectively, blue line), BNZ-SNEDDS (50 mg kg^−1^, green line), BNZ-SNEDDS (100 mg kg^−1^, purple line) over 5 days (*n* = 6). Survival at day 30 post-treatment is significant for both BNZ-SNEDDS groups versus the untreated control (*p*:0.0045 and *p*: < 0.0001 for 50 and 100 mg kg^−1^ respectively) and vs. NFX-BNZ-SNEDDS (*p*: 0.0004 and *p* < 0.0001 for 50 and 100 mg kg^−1^ respectively) (Kruskal Wallis test, 95% level of significance). (**C**): Parasitaemia levels of surviving animals during the acute infection period (days 8, 10 post-infection) in BALB/c male mice infected with 10,000 bloodstream trypomastigotes of *T. cruzi*. Mice were randomly split into groups of six to ensure that a 50% difference in parasitic load can be detected with 80% confidence. Mice received five doses of formulations daily starting at day 5 post-infection. Parasitaemia was determined by counting the number of trypomastigotes in 5 μL of fresh blood collected from the tail (means ± SEMs). Groups were treated with blank SNEDDS (white column), NFX (50 mg/kg/day, grey column) and BNZ (50 mg/kg/day, black column), BNZ-SNEDDS (50 mg/kg/day, salmon column), BNZ-SNEDDS (100 mg/kg/day, green column), and NFX-BNZ-SNEDDS (25 and 50 mg/kg/day respectively, blue column).

The median survival of untreated animals was 15–17.5 days (Figure 8A,B) and oral daily administration of NFX and BNZ at 50 mg/kg as previously reported as active doses [23] was able to increase survival to 30 days post-infection for all treated animals (Figure 8A). Using the combined NFZ-BNZ-SNEDDS formulation enabled the halving of the NFZ and BNZ dose required to ensure 30 days post infection survival in two-thirds of treated animals (Figure 8B). All animals treated with BNZ-SNEDDS at a higher BNZ dose (100 mg kg^−1^) as monotherapy survived for the duration of the study showed a median survival of 30 days were used a monotherapy, which was 1.33-fold higher than when BNZ-SNEDDS at 50 mg kg^−1^ were used with a similar 5 days oral administration regime (Figure 8B). The combined NFZ-BNZ-SNEDDS were able to reduce parasitaemia on all tested days in acute experimental models of CD (Figure 8C).

## 4. Discussion

Chagas Disease (CD) treatment relies on the use of BNZ and NFX, both of which were developed over 4 decades ago [22]. In comparison to other therapies currently under research, they remain the most reliable in treating the acute phase and managing the chronic phase. However, this combination of drugs comes with many patients’ compliance issues such as the long duration of treatment and increased frequency of administration as well as safety issues (e.g., cardiotoxicity) and unpredictable treatment outcomes. Thus, developing an oral combination tablet with improved oral bioavailability, targeting RES organs and reduced frequency of administration can significantly improve therapeutic outcomes. Lipid-based drug delivery systems are effective approaches for poorly soluble APIs as they are known to improve oral bioavailability by enabling higher drug loading, increasing the wettability of API, facilitating selective transportation of API, interacting with electrolyte efflux transporters, and maintaining the API in the solubilized state in the GI tract [25]. SNEDDS, isotropic mixtures of oil, surfactants, and co-surfactants, are an easily scalable, cost-effective, orally bioavailable formulation able to easily combine therapies for CD. Adsorbing SNEDDS on solid carriers can enable the production of cost-effective, easily packaged and manufactured solid dosage forms (tablets, granules) for CD patients [25].

QbD was used to optimize the final SNEDDS composition in terms of particle size (<500 nm) to allow for sizes linked to increased oral bioavailability and increased lymphatic uptake [56,57]. The best fitted quadratic model indicated two optimised formulations of which only solution 1 resulted in a nanoparticulate size and comprised of Labrasol: Labrafil M1944 CS: Capryol 90 (50:10.12:39.88 *w*/*w*). The smallest globule size was observed at low levels of Labrafil M1944 (co-surfactant) and higher levels of Capryol 90 (oil phase) resulting in mixtures with an overall HLB of 8 that results in o/w nanoemulsions with low critical micellar concentrations (Figure 3) [51]. Labrafil M1944 CS as a co-surfactant prevents the drug from precipitating in the lumen (Figure 4), while can allow for enhanced mucous permeation leading to increased absorption [58]. Loaded SNEDDS showed a significant lower particle size and higher zeta potential compared to blank SNEDDS (Table 3, Figure 3) possibly due to attraction forces between the drugs (e.g., BNZ), the oil and surfactants. Based on TLC experiments (please see Supplementary Materials Table S3), NFX does not interact with the hydroxyl groups of silica as strongly as BNZ when formulated in SNEDDS, but the elution is retarded when co-formulated in NFX-BNZ-SNEDDS (0.2% and 1% respectively). The high hydroxyl group presence within the pegylated surfactants used can allow for a strong interaction/attraction force between the APIs resulting in a significantly smaller particle size. SNEDDS were able to be loaded with high amounts of both NFX and BNZ (2.5 and 10 mg g^−1^ almost double than previously reported [59,60]), and are concentrations that can be translated preclinically (allowing for 25 mg kg^−1^ and 50 mg kg^−1^ doses respectively).

In vitro lipolysis assays are useful to understand intestinal digestion of lipid-based formulations such as SNEDDS, but they do not account for pre-intestinal digestion (gastric lipolysis) which accounts for 10–25% of total lipolysis. Two phases were achieved during our lipolysis experiments (o/w emulsion and a pellet [fatty acid precipitated as calcium salts]). Given the use of a high HLB co-surfactant (Labrafil M 1944 CS), a quick formation of an o/w emulsion in the aqueous phase is expected. The lack of an oil layer can be explained by the fact that all excipients are digestible by lipases [61] and the fine droplet size and reduced lipid content of SNEDDS. Prepared SNEDDS maintained drug solubilised in the supernatant and available for absorption. However, pellets can contain drugs precipitated in the amorphous form, which can lead to much higher dissolution in comparison to the crystalline form of the drug [62]. Consumption of sodium hydroxide is indicative of lipolysis progress and remained consistent throughout all the studies. The release from NFX-SNEEDS and BNZ-SNEDDS is not different from the release of each drug from combined NFX-BNZ-SNEDDS and reaches a value of 90% of cumulative release within 30 min when loaded in immediate-release capsules. However, the effect of fed or fasted state in oral absorption of NFX and BNZ or combined SNEDDS has not been elucidated in our studies but previously clinical studies were undertaken under fed conditions [63,64].

Adsorption of SNEDDS on solid carriers is a common strategy to yield solid dosage forms, avoiding the need for expensive liquid-filled capsules and prolonging the stability of produced formulations [25,65]. Syloid silicas have a higher bulk density, and improved uniformity in particle size, processing and handling in comparison to fumed silica, while they have a high oil loading capacity and require a low mass of other excipients to yield free-flowing solids. Syloid 244 and XDP 3050 have a highly-developed network of mesopores that allow for a large adsorptive surface area (314 and 320 m^2^/g respectively) and a high pore volume (1.6 and 1.7 mL/g respectively) with Syloid 244 having a low particle size (2.5–3.7 µm) vs. Syloid XDP 3050 (59 µm) [66]. Syloid 244 acts as a glidant (at levels of 0.25–2.0%), which can aid uniform powder flow, as well as a reduced static behaviour leading to improved flowability, content uniformity, structural stability, quicker tabletting due to unique moisture absorption, high resistance to capping lamination and sticking, and reduced friability. Syloid 3050 possesses a high adsorption capability of liquid per unit volume without compromising other tabletting parameters making it an optimal carrier for oily liquids. High loading of SNEDDS was achieved with final formulations (2:1 *w*/*w*) which allowed clinically translatable amounts of both NFX and BNZ to be loaded. However, Syloid 3050 resulted in powders with better flow (please see Supplementary Materials Table S2) that were further taken into tabletting.

NFX-BNZ-SNEDDS Syloid 244 FP solid SNEDDS demonstrated a higher % of cumulative release for both drugs compared to the release of each drug from NFX- SNEDDS or BNZ-SNEDDS at the same ratio (2:1 *w*/*w*). This can be explained by stronger interactions of drug impregnated SNEDDS to Syloid 244 FP when each drug-loaded SNEDD is loaded individually that are abolished when drugs are present together potentially due to an intramolecular interaction (hydrogen bond) between BNZ and NFX allowing for higher affinity for the surface as also supported by TLC experiments (please see Supplementary Materials Table S3). However, the opposite was observed for NFZ-BNZ-SNEDDS loaded on Syloid XDP 3050, where release from combined NFX-BNZ-SNEDDS is hindered indicating that other interactions (van der Walls) are predominant. BNZ release from BNZ loaded SNEDDS on both Syloid silicas is hindered compared to BNZ loaded SNEDDS alone due to hydrogen bonding with silanol/hydroxyl group and this is more significant for BNZ compared to NFX. Immediate release was observed from compressed tablets that were significantly higher than that achieved from commercial tablets of both drugs under our testing conditions (Figure 6D). Final tables were friable and did not conform to BP specifications and the method applied to overcoat tablets with Eudragit polymers was unsuccessful and thus not able to control the release.

Accelerated stability studies indicated that both NFX and BNZ followed a first order degradation kinetics in both liquid and solid SNEDDS formulations. The activation energy for solid SNEDDS was higher than for liquid SNEDDS (Table 5). BNZ showed enhanced stability compared to NFX and the stability of solid NFX-SNEDDS was over half a year in blister packs under refrigerated conditions. The stability of NFX was further improved when combined with BNZ both in the liquid and the solid formulations exhibiting an increase of 2–3 fold in shelf-life. The BNZ-SNEDDS were stable under refrigerated conditions for over a year and that doubled for solid BNZ-SNEDDS. Physicochemical interactions between NFX and BNZ and interactions with the silanol groups exposed in both mesoporous silicas are responsible for the enhanced stability, while Syloid silicas act as effective desiccants increasing the stability of moisture-sensitive APIs [67].

NFX and BNZ SNEDDS showed enhanced in vitro efficacy in comparison to drugs alone and reduced IC_50_ than those reported for the drugs in literature (NFX: 2.25 ± 0.34 to 5.05 ± 1.70, and BNZ: 9.34 ± 0.78 to 27.30 ± 1.81 µg mL^−1^). Both NFX and BNZ liquid SNEDDS illustrated high epimastigote killing with low IC_50_ values <2 μg mL^−1^. SNEDDS enhanced the solubilisation capacity of NFX and BNZ in media and release was quick explaining their high in vitro efficacy. On the other hand, solid SNEDDS prepared with Syloid 3050 illustrated a lower IC_50_ than Syloid 244 in both epimastigotes and amastigotes, which further indicates their potential to translate into optimal formulations for the treatment of CD. The combined NFX-BNZ-SNEDDS formulation enabled dose reduction for combined SNEDDS required to ensure 30 days post infection survival in treated animals (Figure 8B) and reduce parasitaemia at all tested days in acute experimental models of CD (Figure 8C). Toxicity of blank SNEDDS cannot be excluded potentially due to effects of surfactants in infected animals as the parasite infects the gastrointestinal tract in the acute phase (Figure 8B), however, further histopathological studies are needed to provide conclusive evidence, and this could potentially be mitigated by the use of solid SNEDDS. Further studies are needed to support the use of NFX-BNZ-SNEDDS at lower than 25 and 50 mg/kg/day doses.

## 5. Conclusions

Prepared SNEDDS enhance the solubilisation capacity of NFX and BNZ and provide an easy method to combine them into oral liquid and solid clinically translatable formulations with a predicted shelf-life of 1 and 2 years respectively under refrigeration. NFX-BNZ-SNEDDS with low levels of NFX and BNZ show excellent selectivity for *T. cruzi* compared to fibroblasts and show a favourable target product profile for clinical translation while they were able to increase survival and reduce parasitaemia in a murine model of acute infection.

## Figures and Tables

**Table 1:** Design matrix for the formulation of SNEDDS of Nifurtimox as per the D-optimal mixture design.

	Critical Material Attributes			Critical Quality Attributes
Formulation Code	X1: Labrasol (%)	X2: Labrafil (%)	X3: Capryol 90 (%)	Particle Size after 1/400 Dilution (nm)
**1**	30.0	30.0	40.0	539 ± 255
**2**	50.0	14.8	35.2	497 ± 68
**3**	50.0	19.9	30.1	962 ± 439
**4**	40.2	20.0	39.8	1485 ± 534
**5**	50.0	30.0	20.0	1619 ± 1869
**6**	50.0	19.9	30.1	810 ± 328
**7**	45.1	14.9	40.0	882 ± 310
**8**	40.0	30.0	30.0	823 ± 262
**9**	43.4	23.3	33.3	629 ± 153
**10**	50.0	10.0	40.0	557 ± 97
**11**	40.0	30.0	30.0	278 ± 96
**12**	40.0	30.0	30.0	893 ± 457
**13**	46.6	26.5	26.7	1060 ± 432
**14**	36.8	26.6	36.6	516 ± 236
**15**	30.0	30.0	40.0	764 ± 183
**16**	40.2	20.0	39.8	1397 ± 489

**Table 2:** Solid SNEDDS formulations prepared.

Formulation	Weight (g)						Total (g)
	Blank SNEDDS	NFX SNEDDS (2 mg/g)	BNZ SNEDDS (10 mg/g)	NFX & BNZ SNEDDS (2 & 10 mg/g)	Syloid 244 FP	Syloid XDP3050	
SS1	2				1		3
SS2	2					1	3
NSS1		2			1		3
NSS2		2				1	3
BSS1			2		1		3
BSS2			2			1	3
NBSS1				2	1		3
NBSS2				2		1	3

**Table 3:** Mean particle size, polydispersity, and zeta potential of prepared batches of blank and drug-loaded SNEDDS (*n* = 3).

Formulation	Particle Size (nm)	Polydispersity	Zeta Potential (mV)
Blank SNEDDS	681 ± 76	0.627 ± 0.065	−25.0 ± 0.9
NFZ—SNEDDS (0.2%)	150 ± 20	0.637 ± 0.081	−26.0 ± 0.6
BNZ—SNEDDS (1%)	109 ± 11	0.728 ± 0.045	−25.6 ± 0.7
NFZ—BNZ -SNEDDS (0.2–1%)	132 ± 7	0.610 ± 0.056	−33.1 ± 2.4

**Table 4:** Solid SNEDDS drug loading.

Solid SNEDDS	NFX (mg/g)	BNZ (mg/g)
NSS1	2.664 ± 0.325	-
NSS2	3.116 ± 0.875	-
BSS1	-	5.562 ± 0.621
BSS2	-	4.345 ± 0.432
NBSS1	2.175 ± 0.124	5.925 ± 0.412
NBSS2	1.508 ± 0.445	4.175 ± 0.234

**Table 5:** Shelf-life prediction based on first order degradation fitted to accelerated stability data for liquid and solid SNEDDS (NFX SNEDDS, NSS1, NSS2, NFX and BNZ SNEDDS, NBSS1, NBSS2) over five temperatures (25, 40, 60, 70, 80 °C).

Formulation	Drug	Ea (Kcal mol^−1^)	Shelf-Life at 4 °C & 0% RH (Years)	Shelf-Life at 25 °C & 0% RH (Years)
NFX-SNEDDS	NFX	23.047 ± 3.647	0.423	0.019
NFX-SNEDDS on Syloid 244 FP	NFX	23.486 ± 3.631	0.586	0.028
NFX-SNEDDS on Syloid 3050 XDP	NFX	22.549 ± 3.858	0.682	0.038
NFX-BNZ-SNEDDS	NFX	27.669 ± 4.148	0.942	0.082
NFX-BNZ-SNEDDS	BNZ	17.556 ± 4.813	1.639	0.171
NFX-BNZ-SNEDDS on Syloid 244 FP	NFX	20.911 ± 5.490	1.407	0.282
NFX-BNZ-SNEDDS on Syloid 244 FP	BNZ	23.761 ± 4.551	2.231	0.114
NFX-BNZ-SNEDDS on Syloid 3050 XDP	NFX	26.672 ± 7.764	1.994	0.188
NFX-BNZ-SNEDDS on Syloid 3050 XDP	BNZ	26.626 ± 4.561	> 3	0.162

**Table 6:** Trypanocidal activity of NFX and BNZ formulations on extracellular *T. cruzi* forms and cytotoxicity on fibroblast and macrophages cell lines.

Formulations	Epimastigotes IC_50_ (µg mL^−1^)	NCTC929 Fibroblasts CC_50_ (µg mL^−1^)	J774 Murine Macrophages CC_50_ (µg mL^−1^)	E.S.I. NCTC929	E.S.I. J774 Murine
Nifurtimox	2.00	88.30	95.50	44	48
Benznidazole	11.80	165.50	153.30	14	13
NFX-SNEDDS	0.02	0.12	0.16	6	5
NFX-SNEDDS Syloid 244 FP	0.60	0.70	0.43	1	1
NFX-SNEDDS Syloid 3050 XDP	0.40	1.30	0.29	3	1
NFX-BNZ-SNEDDS	0.03	0.80	0.48	28	17
NFX-BNZ-SNEDDS Syloid 244 FP	0.30	1.50	0.68	5	2
NFX-BNZ-SNEDDS Syloid 3050 XDP	0.20	0.80	0.43	4	2
Key; IC_50_: the concentration needed to inhibit 50% growth of the parasite, CC_50_: the concentration needed to inhibit 50% growth of cultured cells, E.S.I.: Epimastigotes Selectivity Index i.e., CC_50_/IC_50._					

**Table 7:** Antiamastigote activity of NFX and BNZ formulations on *T. cruzi* infected NCTC929 fibroblasts.

Formulations	Concentration (µg mL^−1^)	%AA	±SD
Nifurtimox	10	85	3.6
NFX-SNEDDS	0.08	30	0.12
NFX-SNEDDS Syloid 244 FP	1.3	43	0.7
NFX-SNEDDS Syloid 3050 XDP	1.3	25	1.3
NFX-BNZ-SNEDDS	0.1	95	0.8
NFX-BNZ-SNEDDS Syloid 244 FP	1.3	33	1.5
NFX-BNZ-SNEDDS Syloid 3050 XDP	1.3	36	0.8
Key; %AA: the percentage of growth inhibition of intracellular amastigotes, ±SD: standard devia-tion.			

## Data Availability

Not applicable.

## Supplementary Materials

The following supporting information can be downloaded at: https://www.mdpi.com/article/10.3390/pharmaceutics14091822/s1, Reference [68] are cited in the supplementary materials. Figure S1: Appearance of blank SNEDDS adsorbed on mesoporous silica at different ratios as per Table S1; Figure S2: Tablets prepared by direct compression of sieved granules of blank SNEDDS adsorbed on Syloid 244 (SS1) or blank SNEDDS adsorbed on Syloid XDP 3050 (SS2) or NFX and BNZ SNEDDS loaded on Syloid XDP 3050 (NBSS2) mixed with microcrystalline cellulose (Avicel PH200) and croscarmellose sodium (Primellose) at 5:4:1 *w*/*w* ratio; Table S1: Solid SNEDDS prepared to identify optimum ratio of SNEDDS loading; Table S2: Summary table of Carr’s Index, Hausner ratio and angle of repose for solid SNEDDS (*n* = 3); Table S3: Summary table of Rf values of TLC eluted samples; Table S4: Mathematical models of the obtained experimental data produced by multiple linear regression analysis (MLRA); Table S5: Goodness of fit of the experimental data to the selected model.
