# Supplementary material for: Solid Nanomedicines of Nifurtimox and Benznidazole for the Oral Treatment of Chagas Disease
Source: Pharmaceutics. 2022 Aug 29;14(9):1822. doi: 10.3390/pharmaceutics14091822 (PMC9504116; doi:10.3390/pharmaceutics14091822)
Supplement: Supplementary file 1 [file pharmaceutics-14-01822-s001.zip › pharmaceutics-1874945-supplementary.pdf]

Supplementary Information
Solid Nanomedicines of Nifurtimox and Benznidazole for the Oral Treatment of Chagas Disease

Miriam Rolon[a*†], Eustine Hanna[b†], Celeste Vega[a], Cathia Coronel[a], Maria-Auxiliadora Dea-Ayuela[c], Dolores R. Serrano[d*], Aikaterini Lalatsa[b,e*].

[a]Centro para el Desarrollo de la Investigacion Científica (CEDIC), Manduvirá 635 entre 15 de Agosto y O'Leary, 1255 Asuncion, Paraguay

[b]Biomaterials, Bio-engineering and Nanomedicines (BioN) laboratory, Institute of Biomedical and Biomolecular Sciences, School of Pharmacy and Biomedical Sciences, University of Portsmouth, White Swan Road, Portsmouth, PO1 2DT, UK.

[c]Departamento de Farmacia, Facultad de Ciencias de la Salud, Universidad CEU Cardenal Herrera, Edificio Seminario s/n, 46113-Moncada, Valencia, Spain

[d]Departament of Pharmaceutics and Food Technology and Instituto Universitario de Farmacia Industrial (IUFI), School of Pharmacy, University Complutense de Madrid, Plaza Ramón y Cajal s/n, 28040, Madrid, Spain

[e]School of Pharmacy and Biomedical Sciences, John Arbuthnot Building, Robertson Wing, University of Strathclyde, 161 Cathedral Street, Glasgow, G4 0RE, UK.

[†]Miriam Rolon and Eustine Hanna share first co-authorship

*Authors for correspondence

Dr. Aikaterini Lalatsa, Reader in Nanomedicines, School of Pharmacy and Biomedical Sciences, John Arbuthnot Building, Robertson Wing, University of Strathclyde, 161 Cathedral Street, Glasgow, G4 0RE, UK, Email: aikaterini.lalatsa@strath.ac.uk, Tel: +44 141 548 2675.
Dr Dolores R. Serrano Lopez, Associate Professor, Departament of Pharmaceutics and Food Technology and Instituto Universitario de Farmacia Industrial (IUFI), School of Pharmacy, University Complutense de Madrid, Plaza Ramón y Cajal s/n, 28040, Madrid, Spain, Email: dr.serrano@farm.ucm.es, Tel: +34913941620

SI – 1.0 Preparation of drug loaded solid SNEDDS.

To prepare solid SNEDDS, drug loaded SNEDDS were adsorbed on mesoporous silica (Syloid 244 FP and Syloid XDP 3050) using a mortar and pestle and gentle mixing. Blank SNEDDS were loaded initially with different weight ratio on both silicas to produce SNEDDS: silica 1:1, 1:2, 1:3, 2:1 and 3:1 w/w ratios and the angle of repose, Carr's index and Hausner ratio was measured (British Pharmacopoeia BP 2020, Volume V, Appendix XVII N) [68]. Angle of repose was measured for 2g of powder using a plastic powder funnel (ø: 120 mm, bore: 20mm) that was fastened 2 cm from the base. When the tap was removed the diameter of the symmetrical powder cone formed on graph paper was measured. Angle of repose was calculated using the following equation:

$$\tan(a) = \frac{height\ (h)}{0.5\ diameter\ of\ cone\ (D)} \qquad \text{Equation SI1.1}$$

where h is the height of the powder cone (in cm) and D is the diameter of the powder cone (in cm) and a is the angle of repose (in degrees). The bulk density (unsettled apparent volume Vo of solids) (2g) was measured in a 30 mL glass cylinder, after which the cylinder was raised and tapped on a hard surface 100 times recording the volume at the end (Vf, tapped density) and the Carr's Index and Hausner ratio was calculated using the following equations:

$$Compressibility\ Index = 100\ \frac{Vo-Vf}{Vo} \qquad \text{Equation SI1.2}$$

$$\text{Hausner Ratio} = 100\ \frac{Vo}{Vf} \qquad \text{Equation SI1.3}$$

Based on the Carr Index, Hausner ratio, angle of repose (Table S2), SNEDDS adsorbed on Syloid 244 FP illustrated better flowability even at high loads of SNEDDS (SN:S7). Increasing the amount of liquid SNEDDS adsorbed on silica up to 2:1 ratio resulted in an increase in compressibility. Solids SNEDDS based on Syloid XDP 3050

up to 2:1 ratio illustrated optimal angle of repose and thus were selected for compression and tableting.

Table S1. Solid SNEDDS prepared to identify optimum ratio of SNEDDS loading.

Formulation	Weight (g)			Total (g)
	Blank SNEDDS	Syloid 244 FP	Syloid XDP3050	
SN:S1	1	3	-	4
SN:S2	1	2	-	3
SN:S3	1	1	-	2
SN:S4	1	-	3	4
SN:S5	1	-	2	3
SN:S6	1	-	1	2
SN:S7 or SS1	2	1	-	3
SN:S8 or SS2	2	-	1	3
SN:S9	3	1	-	4
SN:S10	3	-	1	4

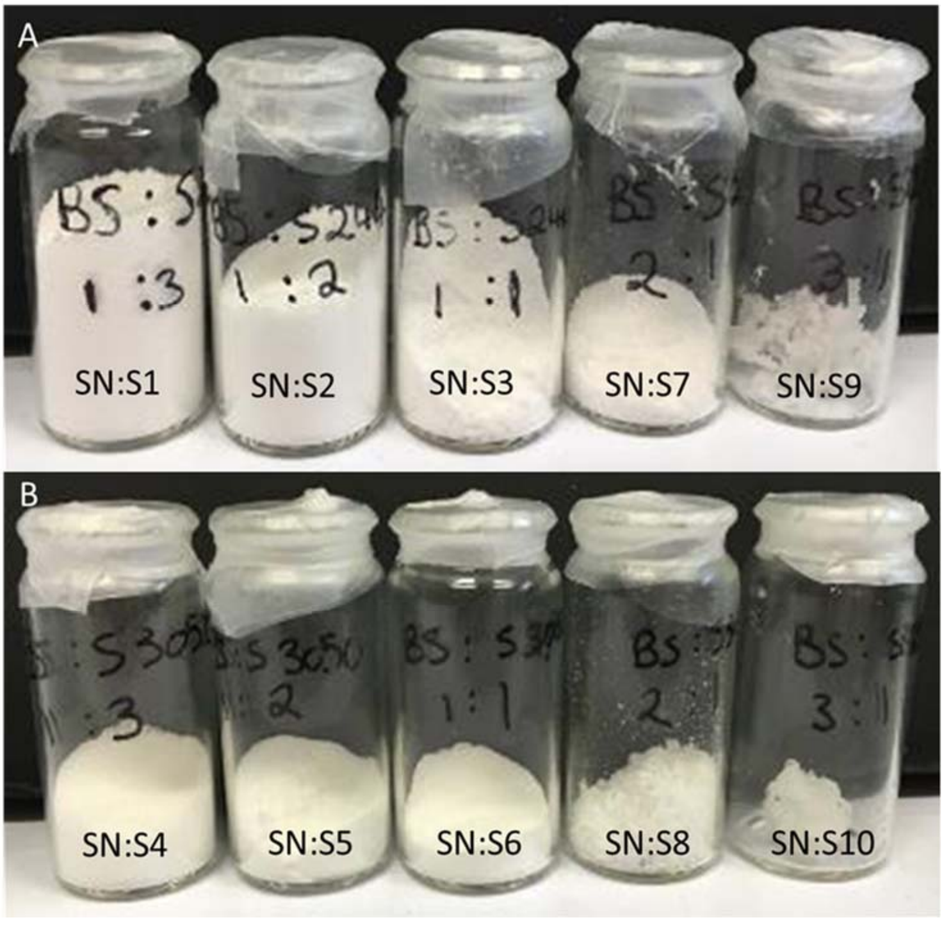

Figure S1. Appearance of blank SNEDDS adsorbed on mesoporous silica at different ratios as per Table S1.

Table S2. Summary table of Carr's Index, Hausner ratio and angle of repose for solid SNEDDS (n=3).

Tested Ratio	Carr's Index	Hausner Ratio	Angle (°) of Repose	Flowability
SN:S1	17.83 ± 1.73	1.22 ± 0.028	23.89 ± 1.89	Fair
SN:S2	19.44 ± 0.01	1.24 ± 0.04	27.70 ± 0.66	Good
SN:S3	22.22 ± 0.02	1.22 ± 0.02	27.29 ± 0.51	Good
SN:S4	18.18 ± 0.01	1.19 ± 0.009	10.65 ± 2.80	Fair – Passable
SN:S5	16.24 ± 0.61	1.22 ± 0.01	13.71 ± 1.48	Fair – Passable
SN:S6	20.36 ± 2.35	1.26 ± 0.042	14.74 ± 0.87	Passable
SN:S7	15.82 ± 3.33	1.21 ± 0.009	25.97 ± 2.28	Good
SN:S8	33.33 ± 0.50	1.5 ± 0.01	30.03 ± 2.85	Passable
SN:S9	*	*	*	*
SN:S10	*	*	*	*

* : Could not be assessed as not a powder

SI – 2.0 Preparation of tablets from solid SNEDDS after wet granulation.

A compression force of 10 Newton was used to prepare batches of SS1 and SS2 granules and drug loaded solid SNEDDS granules using both mesoporous silicas. Figure S2 illustrates the appearance of produced batches.

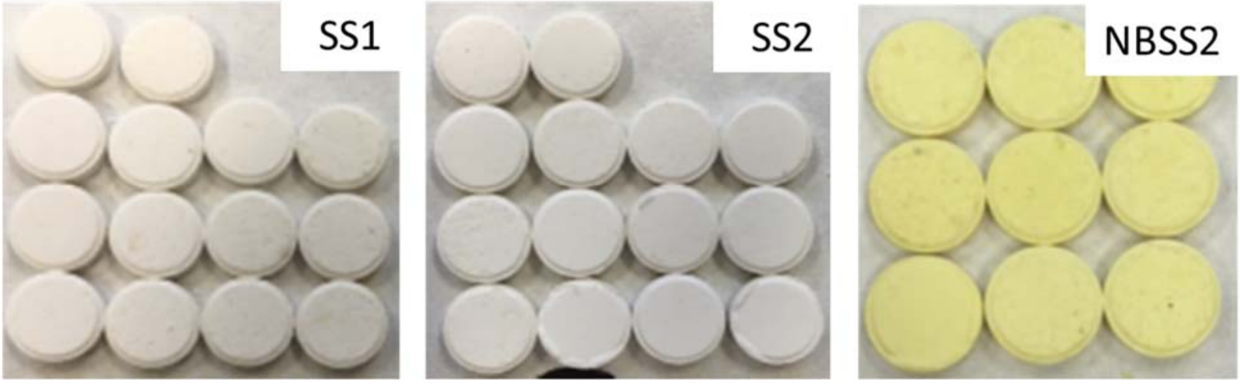

Figure S2. Tablets prepared by direct compression of sieved granules of blank SNEDDS adsorbed on Syloid 244 (SS1) or blank SNEDDS adsorbed on Syloid XDP 3050 (SS2) or NFX and BNZ SNEDDS loaded on Syloid XDP 3050 (NBSS2) mixed with microcrystalline cellulose (Avicel PH200) and croscarmellose sodium (Primellose) at 5:4:1 w/w ratio.

SI – 3.0 TLC analysis of formulations

NFX (0.2% in dichloromethane), BNZ (1% in dichloromethane), blank SNEDDS, NFX-SNEDDS (0.2%), BNZ-SNEDDS (1%) and NFX-BNZ-SNEDDS (0.2% and 1% respectively) were spotted on a drawn baseline 1cm from the bottom of a 6.5 cm long aluminium backed pre-coated TLC sheet (ALUGRAM SIL G/UV254, 0.2mm, silica gel 60 with fluorescent UV indicator 254 nm, Macherey-Nagel GmbH & Co KG, Duren, Germany). Plate was inserted in a TLC jar pre-saturated with dichloromethane (mobile phase). The solvent front was immediately drawn when the solvent front reached ¾ of the length of the plate and when dried was visualised under UV light (254 nm, Model UVGL-58 Mineralight© lamp, Multiband UV 254/365 nm, UVP, Upland, CA, USA). The Rf value was calculated by dividing the distance travelled in cm of the sample spots vs the distance travelled of the solvent front from baseline (Table S3).

Table S3. Summary table of Rf values of TLC eluted samples.

Compound/Formulation	Rf values
NFX	0.41
BNZ	0.11
Blank SNEDDS	0.58
NFX-SNEDDS	0.41, 0.59
BNZ-SNEDDS	0.11, 0.59
NFX-BNZ-SNEDDS	0.09, 0.59

SI – 4.0 Quality by Design (QbD) studies for optimising NFX and BNZ SNEDDS; selection of optimal mathematical models for linking ratio of oils and surfactants to desired CQA of SNEDDS.

A quadratic fitting model was the optimal mathematical model for linking ration of GRAS oils and surfactants to the desired CQA for SNEDDS (Table S4). The co-efficient of the models generated for CQAs (Table S5) revealed medium goodness of fit of the experimental data to the selected model but able to be used to navigate the design space (R^2: 0.74, low values of PRESS, a negative predicted R-squared which indicates that the overall mean may be a better predictor of droplet size in the current model and a ratio greater than 4 for the adequate precision indicative of signal to noise ratio).

Table S4. Mathematical models of the obtained experimental data produced by multiple linear regression analysis (MLRA).

**Source**	**Sequential p-value**	**Lack of Fit p-value**	**Adjusted R^2**	**Predicted R^2**	
Linear	0.6194	0.0698	-0.0719	-0.4926	
Quadratic	**0.0042**	**0.4543**	0.6068	-0.0178	**Suggested**
Special Cubic	0.1523	0.5603	0.6565	-0.0127	
Cubic	0.4583	0.4884	0.6554	-3.2113	
Special Quartic vs Quadratic	0.4063	0.4078	0.6196		
Quartic vs Cubic	0.4884		0.6280		Aliased
Quartic vs Special Quartic	0.4078		0.6280		Aliased

Table S5. Goodness of fit of the experimental data to the selected model.

Standard Deviation	0.24		R^2	0.7379
Mean	0.86		Adjusted R^2	0.6068
Coefficient of Variation C.V.%	27.75		Predicted R^2	-0.0178
PRESS	2.20		Adequate Precision	8.152
